# Effect of an Eight Week Dietary Intervention of Pecans on Cardiometabolic Parameters and Serum Cytokines in Individuals with Obesity or Overweight: A Preliminary Study

**DOI:** 10.3390/cimb48080798

**Published:** 2026-08-06

**Authors:** Samuel McCormick, Tessa Solberg, James Scoles, Christopher Mayne, Maria Morgan-Bathke

**Affiliations:** 1 Nutrition and Dietetics Department, Viterbo University, La Crosse, WI 54601, USA; 2 Biology Department, Viterbo University, La Crosse, WI 54601, USA

**Keywords:** obesity, MUFA, pecan, cardiometabolic health, inflammatory markers, dietary intervention, nutrient intake

## Abstract

Obesity is a major health concern within the United States. Previous studies have shown that a diet high in monounsaturated fatty acids (MUFA) may help combat metabolic disease by improving cardiometabolic and inflammatory status. Pecans have high MUFA content and the highest antioxidant capacity of any nut. The aim of this preliminary study is to determine if a diet supplemented with 1.5 ounces of pecans daily for eight weeks could improve blood glucose, lipid panel, inflammatory markers, and body composition in individuals with overweight and obesity as compared to a control group who maintained their normal diet. Post-intervention measurements showed no significant difference from baseline values for either the experimental or the control group. Data acquired using the Vioscreen Food Frequency Questionnaire found negative correlations were present between LDL cholesterol and dietary intake of iron, selenium, zinc, MUFA, polyunsaturated fatty acids, saturated fatty acids, and dietary cholesterol. Furthermore, there were negative correlations determined between fasting blood glucose and dietary intake of magnesium, MUFA, and SFA. Regarding inflammatory markers, no detectable levels of IL-10 nor TNF-α were found in the majority of samples. IL-6 was detectable in all samples, but showed no statistical difference between any of the groups in the study. Thus, an intervention of 1.5 oz of pecans for eight weeks did not significantly change measured metabolic parameters nor levels of serum IL-6. However, due to the sample size limitations of this hypothesis-generating preliminary study, further research is needed. Further studies should include a longer intervention period, a larger cohort, and potentially a larger dosage of pecans to increase the amount of MUFA in the diet to determine if such interventions may influence cardiometabolic and inflammatory parameters.

## 1. Introduction

Obesity is a major health concern worldwide. According to the World Health Organization, 890 million adults are living with obesity. Obesity is defined as an abnormal amount of accumulated adipose tissue to the extent of hindering health [1]. Obesity is associated with an increased risk of comorbidities including cardiovascular diseases, gall bladder disease, hyperuricemia, and osteoarthritis. Obesity is also a major risk factor contributing to the development of type 2 diabetes mellitus [2]. The number of individuals with insulin resistance and dysregulation of blood glucose homeostasis has been rising in the United States at an alarming rate. As of 2023, there were approximately 29 million U.S. citizens diagnosed with type 2 diabetes mellitus (T2DM), and an estimated 11 million citizens who are undiagnosed [3]. Additionally, about 115 million, or greater than 4 in 10 adults in the U.S., are pre-diabetic [3]. Obesity has also been correlated to differences in the levels of proinflammatory and anti-inflammatory cytokines. For example, individuals with obesity tend to express higher levels of systemic TNF-α [4].

Cell culture studies, animal models, and epidemiological research have indicated that there may be a positive association between increased proportions of monounsaturated fatty acid (MUFA) intake and improved blood glucose and triglyceride levels, suggesting the potential for an easily accessible dietary intervention [5,6,7]. Monounsaturated fatty acids are found in foods such as vegetables, nuts, meats, dairy and seed oils. They are defined as containing one double bond, generally located at carbon nine from the methyl end of the fatty acid hydrocarbon chain [8]. Oleic acid is the most abundant MUFA found in foods, thus making it the most abundantly consumed in the American diet [8]. Although there is not an accepted macronutrient distribution range for fatty acids, it is generally recommended that saturated fatty acids (SFA) are replaced with MUFA and PUFA [8].

MUFA intake has been linked to increasing HDL while reducing LDL, triglycerides, and total cholesterol to HDL ratio [8,9,10]. Clinical studies have shown a change in body composition with an increase in consumption of monounsaturated fatty acids. A study conducted by Piers et al. investigated the influence of an average Western diet compared to one that was rich in SFA and another that was rich in MUFA [11]. The results showed a significantly higher body weight after the SFA-rich diet than the MUFA-rich diet [11]. Moreover, percent body fat, trunk fat mass, and limb fat mass were also significantly greater after the SFA-rich diet [11]. In another study conducted by Paniagua et al. it was discovered that an isocaloric MUFA-rich diet prevented central fat redistribution [12]. Furthermore, previous studies have shown benefits of both MUFA and micronutrients on serum lipid and lipoprotein profiles. One study conducted by Farvid et al. assessed the impact of magnesium, zinc and vitamins on patients with type 2 diabetes [13]. It was discovered that after 3 months of co-supplementation, serum levels of HDL-c and apo A1 increased significantly [13]. Other studies indicate that MUFA and the micronutrients iron, magnesium, and zinc may reduce insulin resistance [14,15].

Dietary fatty acid intake also appears to correlate to levels of blood cytokines, potentially indicating an effect on inflammatory status [16]. Proinflammatory TNF-α was found to be at increased levels in plasma of higher SFA to MUFA ratio diets compared to diets with a lower SFA to MUFA ratio [17]. A diet higher in SFA also showed a statistically significant increase in plasma IL-6 levels over a diet that had less SFA and more MUFA in a cohort of young women [18]. It has been suggested that SFA leads to a heightened immune response due to mimicking lipopolysaccharide (LPS), which is a common immune-activating molecule found in Gram-negative bacteria [19].

Tree nuts have been emphasized as an important addition to Americans’ diets as their fatty acid profile is high in MUFA and PUFA [20]. Furthermore, tree nuts contain fiber, phytosterols, polyphenols, vitamins and minerals that all contribute to a healthful diet [20]. Pecans are considered to be a traditional tree nut in the United States; however, there is not a clear recommendation of the consumption amount necessary to provide improvements in health [20]. Because individuals with pre-diabetes have an abnormal fasting blood glucose level, and individuals with obesity are prone to insulin resistance, these two groups are an ideal population to test the efficacy of increased MUFA intake on improving blood glucose, triglycerides, and body composition for the prevention of T2DM. Studies have shown evidence to support that consumption of nuts can lower the overall risk of cardiovascular disease (CVD) in patients that already have T2DM and higher risk for CVD is correlated with higher levels of inflammatory cytokines such as IL-6 [21].

This preliminary study aims to investigate the effect of an eight-week dietary intervention of pecans on fasting blood glucose, lipid profile, body composition, and serum cytokines in individuals that have pre-diabetes and/or overweight/obesity. The purpose of the study is to generate hypotheses with the aim of conducting larger scale studies in the future. Study participants were tested for blood lipids, serum cytokine levels, waist-to-hip ratio (WHR), height, weight, body mass index (BMI), and visceral body fat. Participants also completed a food frequency questionnaire using VioCare to determine average dietary intake and further explore links between diet, lipid profiles, cardiometabolic parameters, and serum cytokine levels.

## 2. Materials and Methods

### 2.1. Participants

This hypothesis-generating study (ClinicalTrials.gov registration number: NCT07732816) was approved by the Viterbo University IRB committee (IRB Number: 1189; date: 16 November 2016). This study was conducted in accordance with the principles of the Declaration of Helsinki. All participants provided written informed consent prior to enrollment. Patient recruitment and follow-up were conducted between October 2017 and October 2018. Inclusion criteria included: adults, ages 18–65 years, with a BMI of 25.0–40.0, which signifies they have overweight or obesity. Exclusion criteria included: anyone who works a 3rd shift, pregnant and breastfeeding women, those with pre-existing chronic conditions, and those with a latex allergy. The demographics of the participants were: 22 participants, of which 18 were female and four were male. Three individuals were excluded from the study due to being unable to obtain all measurement requirements. The average age of the participants was 49 with a standard deviation of 3.8. Participant characteristics are outlined in Table 1.

### 2.2. Dietary Intervention

Participants were randomly placed into either a pecan intervention or control group using a random number table. Participants in the pecan group received 1.5 oz pre-portioned packs of roasted, unsalted pecans. This serving provides about 27 g of MUFA compared to the recommended 33–44 g daily for a standard 2000 calorie diet. All participants consumed the standard nut serving size of 1.5 oz, regardless of body weight. They were instructed to consume 1.5 oz of pecans daily between the hours of 08:00 and 14:00 for eight weeks. These time guidelines were given to ensure consistent measurements since the time in which individuals consume meals can affect cardiometabolic parameters [22]. The participants in the control group were instructed to continue to follow their usual diet, excluding pecans, for the eight weeks.

### 2.3. Sample Collection and Preparation

A registered nurse performed a venous blood draw to obtain the blood samples. Whole blood samples were collected from the study participants into SST vacutainer tubes (BD Biosciences, San Jose, CA, USA) and separated into serum by centrifugation at 1200× *g* for 10 min. Serum samples were split into multiple aliquots to prevent repeated freeze–thaw cycles and then stored at −80 °C until used in later assays.

### 2.4. Blood Lipids and Glucose

Participants were asked to fast 8 h prior to lipid panel and blood glucose tests. Blood was then analyzed using the Cholestech LDX (Abbott, Abbott Park, IL, USA). This was used to get a full lipid panel reading, interpreting the amount of HDL, LDL, VLDL, triglycerides, and total cholesterol.

### 2.5. Serum Cytokine Assays

The enzyme-linked immunosorbent assay (ELISA) used for TNF-α analysis was the Invitrogen Human TNF-α ELISA kit (Invitrogen, Carlsbad, CA, USA). IL-10 was analyzed using the R&D Systems Human IL-10 Quantikine ELISA kit (R&D Systems, Minneapolis, MN, USA). IL-6 was analyzed using the R&D Systems Human IL-6 Quantikine ELISA kit (R&D Systems, Minneapolis, MN, USA). The kit protocols were followed for all three assays. The detection range for the TNF-α ELISA was 1.7 to 1000 pg/mL. The detection range for IL-10 was 3.9 to 500 pg/mL and the range for IL-6 was 0.626 to 300 pg/mL.

### 2.6. Body Composition Assessment

The body composition measurements taken were height, weight, BMI, WHR, and visceral fat. A Maltron BioScan 920-II (Maltron International, Essexm, UK) was used to measure visceral fat and total body fat percentage using electrical impedance and resistance. The visceral fat is reported in ohms, which was imputed, along with abdominal circumference. Waist-to-hip ratio was taken by measuring the waist circumference and hip circumference of each individual and then calculating the ratio by dividing the waist measurement by the hip measurement. Height was measured in meters and weight was measured in kilograms. BMI was calculated using the equation of kilograms/meters^2^.

### 2.7. Food Frequency Questionnaire Assessment

All participants completed the Vioscreen FFQ. FBG and LDL cholesterol were compared with dietary intake of iron, selenium, magnesium, vitamin E, zinc, MUFA, PUFA, and SFA, and dietary cholesterol. Additionally, correlation tests were done between all participants’ BMI, HDL, LDL, VLDL, total cholesterol and blood glucose levels and dietary intake of vitamins A, C and D, sodium and dietary fiber.

### 2.8. Statistical Analysis

After baseline and endpoint measurements, visceral body fat, WHR, BMI, total body fat percentage, and lipid panel and blood glucose values were compared between all participants by averaging the values in each category and completing a Shapiro–Wilk test for normality. Following this, a two-tailed, paired *t*-test was utilized to assess variations between the control group and experimental group. All data is expressed as the mean ± standard error of the mean (SEM). A univariate regression analysis was completed using JMP Pro 15 statistical software program (Cary, NC, USA) to find statistical correlations for the food frequency questionnaire; Bonferroni corrections were not used for these multiple comparisons to avoid the risk of type 1 error. Statistical analysis of the serum cytokine concentrations used two-tailed *t*-tests assuming unequal variances between the pre-treatment groups, along with paired two-tailed *t*-tests comparing pre-intervention and post-intervention groups in both the control and treatment groups. Due to the small sample sizes, a Wilcoxon matched pairs rank test and Mann–Whitney tests were also performed.

## 3. Results

### 3.1. Body Composition

Baseline measurements showed no significant difference between the intervention and control groups in height (*p* = 0.9), weight (*p* = 0.6), BMI (*p* = 0.6), WHR (*p* = 0.5), visceral fat (*p* = 0.3), and body fat percentage (*p* = 0.5). Endpoint measurements showed no significant difference between the control group compared to baseline values BMI (*p* = 0.84), WHR (*p* = 0.56), body fat percentage (*p* = 0.65), and visceral fat (*p* = 0.36) (Table 2). As stated above, a Maltron BioScan 920-II was used to measure visceral fat and total body fat percentage using electrical impedance and resistance. There was no significant difference between endpoint values of the control compared to the pecan group BMI (*p* = 0.55), WHR (*p* = 0.33), body fat percentage (*p* = 0.82), and visceral fat (*p* = 0.69) (Table 3).

### 3.2. Blood Lipids and Glucose

Baseline and endpoint data show no significant difference between participants’ lipid panel and fasting blood glucose (Table 4). Baseline measurements show no significant difference in total cholesterol (*p* = 0.32), HDL (*p* = 0.95), LDL (*p* = 0.35), VLDL (*p* = 0.72), triglycerides (*p* = 0.53), and fasting blood glucose (*p* = 0.67) between the control and experimental group. Endpoint measurements between control and experimental groups also showed no difference in total cholesterol (*p* = 0.23), HDL (*p* = 0.95), LDL (*p* = 0.48), VLDL (*p* = 0.84), triglycerides (*p* = 0.19), and fasting blood glucose (*p* = 0.12). There were no differences within each group at baseline and endpoint (Table 4).

### 3.3. Food Frequency Questionnaire

**Figure 1:**
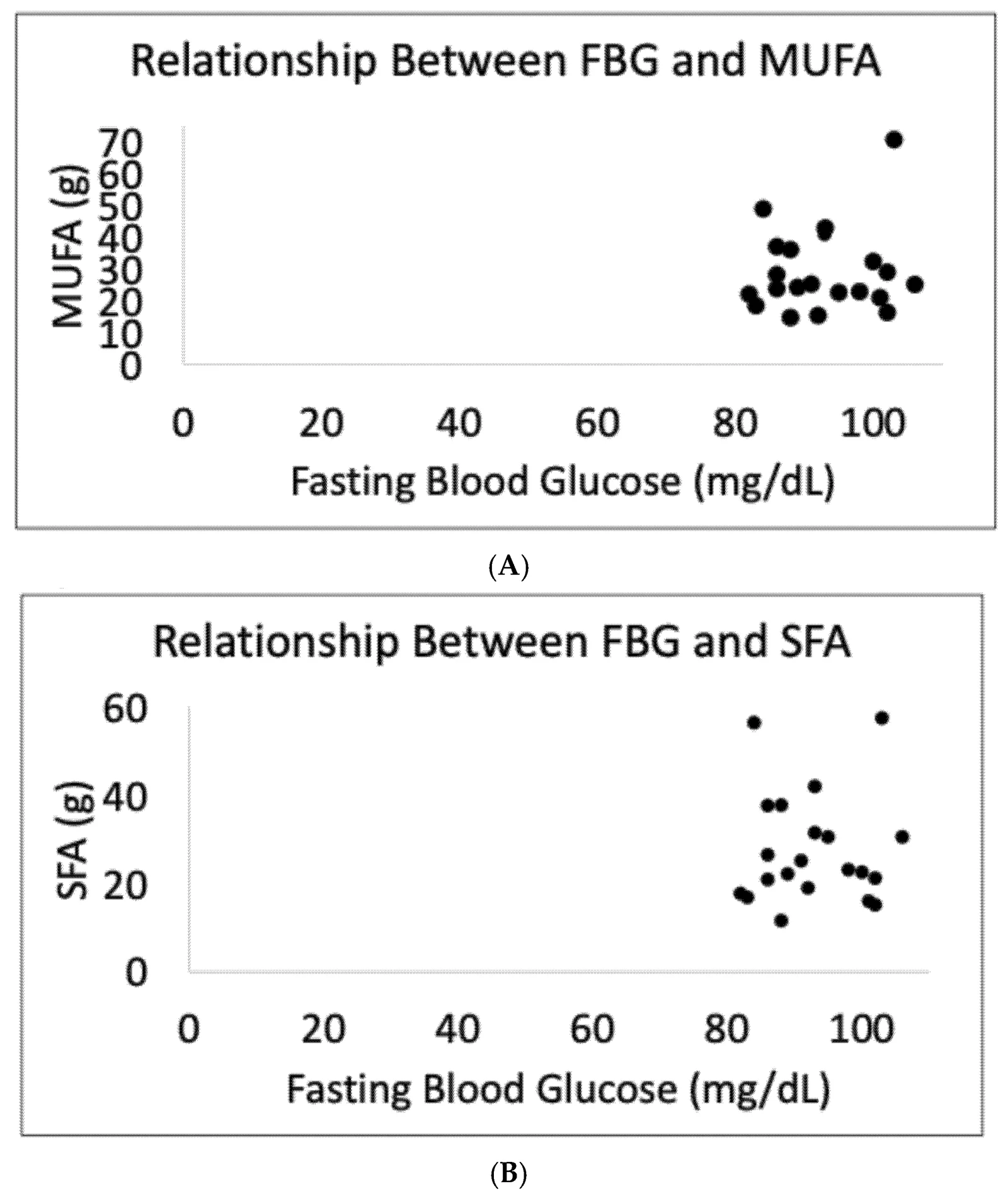
(**A**) displays the relationship between fasting blood glucose and intake of monounsaturated fatty acids; (**B**) displays the relationship between fasting blood glucose and intake of saturated fatty acids.

**Figure 2:**
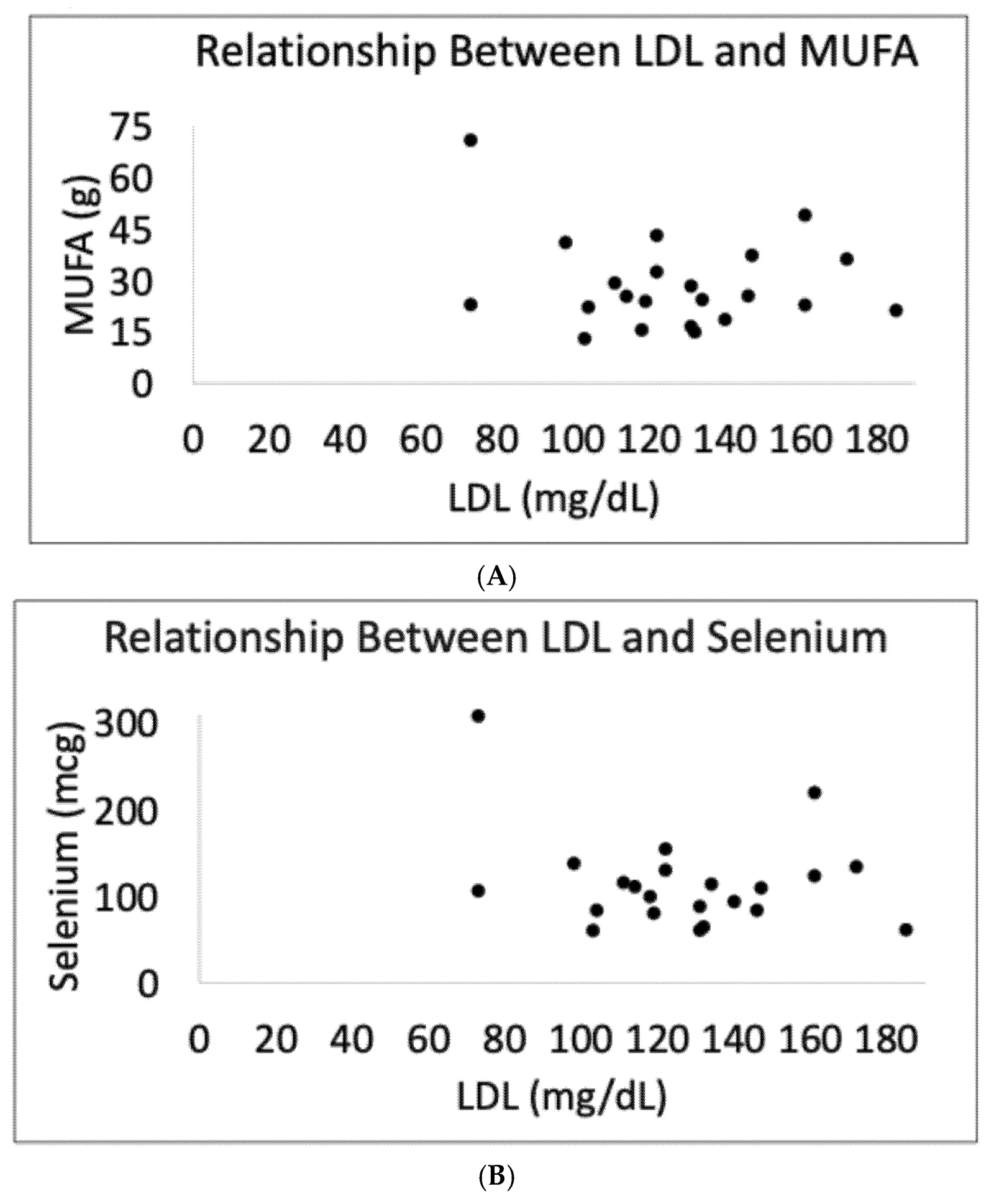
(**A**) displays the relationship between LDL cholesterol and intake of monounsaturated fatty acids; (**B**) displays the relationship between LDL cholesterol and intake of selenium.

Fasting blood glucose (FBG) levels were compared to numerous nutrient values determined by FFQ (Table 5). There were statistically significant negative correlations between FBG and dietary intake of magnesium (*r* = −0.20, *p* = 0.01, very weak), MUFA (*r* = −0.16, *p* = 0.05, Figure 1A, very weak), and SFA (*r* = −0.20, *p* = 0.03, Figure 1B, very weak). LDL levels were compared to numerous nutrient values determined by FFQ (Table 6). There was a statistically significant positive correlation between LDL cholesterol and dietary intake of magnesium (*r =* 0.04, *p* = 0.01, very weak). Statistically significant negative correlations were present between LDL cholesterol and dietary intake of iron (*r* = −0.13, *p* = 0.01, very weak), zinc (*r* = −0.21, *p* = 0.004, very weak), MUFA (*r* = −0.18, *p* = 0.007, Figure 2A, very weak), PUFA (*r* = −0.34, *p* = 0.0001, weak), SFA (*r* = −0.06, *p* = 0.03, very weak), selenium (*r* = −0.25, *p* = 0.004, Figure 2B, very weak), and dietary cholesterol (*r* = −0.34, *p* = 0.02, weak) (Table 6). A significant correlation appeared between vitamin D and BMI (*r* = 0.71, *p* = 0.0004, strong), vitamin D and HDL (*r* = −0.48, *p* = 0.007, weak), vitamin C and VLDL (*r* = −0.44, *p* = 0.04, weak), and sodium and VLDL (*r* = −0.35, *p* = 0.0003, very weak) (Table 7). Other correlations between FFQ-determined micronutrients and cardiometabolic parameters did not have *p*-values less than 0.05, suggesting a lack of statistical significance (Table 7).

### 3.4. Serum Cytokine Analysis

**Figure 3:**
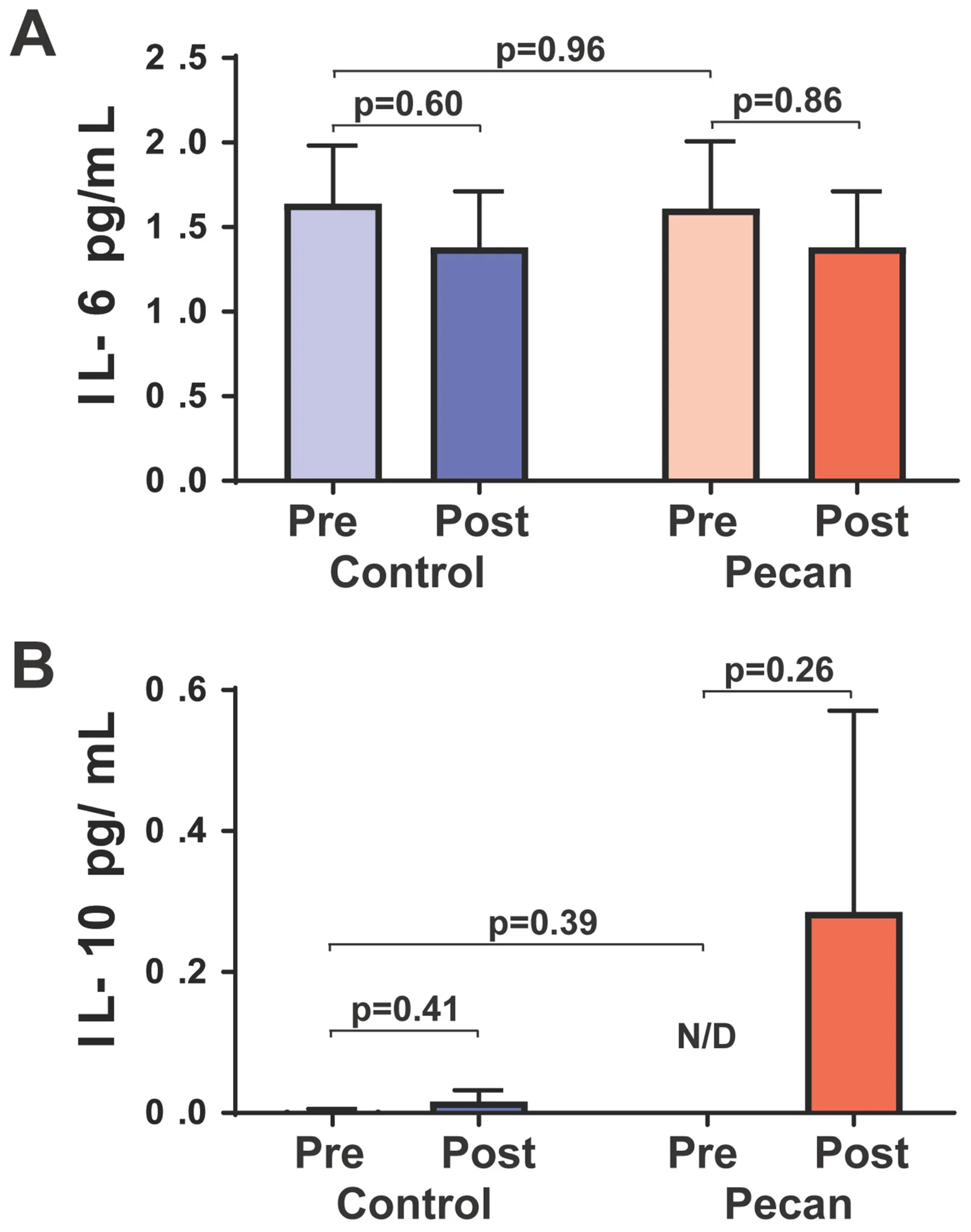
Comparison of mean serum cytokine levels for each experimental group. (**A**) IL-6 cytokine analysis. (**B**) IL-10 cytokine analysis. Sample size for control group preintervention was *n* = 10, post-treatment was *n* = 9. Treatment group sample size pre-intervention was *n* = 8 and post-intervention was *n* = 6. N/D shown for pre-intervention levels of IL-10 since no samples had detectable levels of IL-10 in the pecan supplement group.

IL-6 levels were detectable in all samples that were assayed (detection range 0.626 to 300 pg/mL). The mean concentration of IL-6 for the pre-intervention treatment group was 1.61 pg/mL (*n* = 8), with the pre-intervention control group being 1.64 pg/mL (*n* = 10), and a two-tailed *t*-test yielded *p* = 0.96, suggesting no differences in the groups pre-intervention (Figure 3A). Given the small sample size, a Mann–Whitney test between these groups was also performed and yielded *p* > 0.999, further suggesting no differences pre-intervention. After the intervention, the experimental group showed a mean concentration of 1.72 pg/mL (*n* = 6), and the control group had a mean concentration of 1.38 pg/mL (*n* = 9). IL-6 showed no significant differences between any groups using paired two-tailed *t*-tests with *p* = 0.60 between the controls pre- and post-intervention and *p* = 0.86 between the pre- and post- pecan intervention data (Figure 3A). Given the small sample sizes, non-parametric tests were also performed to fortify the results. Specifically, a Wilcoxon matched pairs rank test gave *p* = 0.82 between the controls pre- and post-intervention and *p* = 0.95 between the pecan group pre- and post-intervention.

IL-10 levels were also not statistically significant between any groups, with a two-tailed *t*-test *p* = 0.39 between pre-intervention groups and paired two-tailed *t*-tests yielding *p* = 0.41 between pre- and post-intervention samples in the control and *p* = 0.26 between pre- and post-intervention samples in the pecan group. Furthermore, a Mann–Whitney test between pre-intervention groups and Wilcoxon matched pairs tests between pre- and post-intervention groups all yielded *p* > 0.99. However, IL-10 levels were generally very low and undetectable in several samples, likely contributing to these results (Figure 3B). TNF-α was also undetectable in 39 of the 40 participants assayed. This could be due to the range of concentrations the regular sensitivity assay detects being too high for the amount of TNF-α (15.6 to 1000 pg/mL) or IL-10 (7.8 to 500 pg/mL) in these samples. No statistically significant differences were calculated from this group of data (Figure 3), and all measured serum cytokine levels were within reported normal ranges (IL-6 ≤ 5.3 pg/mL, IL-10 ≤ 2.9 pg/mL, and TNF-α ≤ 15.4 pg/mL) [23].

## 4. Discussion

In this preliminary study, pecans were used to determine if an eight-week dietary intervention high in MUFA could improve cardiometabolic effects in individuals with overweight or obesity. Upon completion of the study, we found that (1) there were no significant differences in either fasting blood glucose or lipid panel between the control and experimental group post-intervention; (2) there was no significant difference in body composition between the control and experimental groups post-intervention; and (3) there is a significant correlation between some dietary intake values according to FFQ and cardiometabolic parameters. This preliminary study suggests that an eight-week dietary intervention of consuming 1.5 oz of pecans per day does not improve fasting blood glucose, lipid panel values, nor body composition but does suggest that the healthy fats in pecans along with other dietary factors analyzed in the FFQ may improve certain cardiometabolic parameters.

Although we did not find that daily pecan consumption is able to significantly improve lipid panel values, other studies have found that daily nut consumption can improve these parameters. One pooled analysis of 25 nut consumption trials found that a mean daily consumption of 67 g of nuts had the ability to reduce some lipid panel values [24]. Sabate et al. found that there was a mean reduction in total cholesterol, LDL cholesterol concentration, ratio of LDL-C to HDL-C, and there was a reduction in triglyceride levels [24]. According to the findings of this analysis, the improvement of lipid levels is dose-related. In this study, a dose of 1.5 oz was consumed each day which could contribute to the lack of statistical significance, as the 67 g that was used in the pooled analysis is equivalent to 2.36 oz [24]. In another study conducted by Rajaram S et al., pecans were compared to the Step I diet for the effect that they had on blood lipids and lipoproteins in healthy men and women [25]. Although the Step I diet is a cholesterol lowering diet, it was discovered that the pecan-enriched diet altered the lipid profile more favorably than the Step I diet. Furthermore, it was found that the pecan-enriched diet lowered serum cholesterol, increased HDL cholesterol and lowered triacylglycerol concentrations [25].

Similarly, we found no significant difference in body composition between the control group and the experimental group at baseline and endpoint, indicating the consumption 1.5 oz. of pecans per day does not affect BMI, WHR, body fat percentage, nor visceral fat. This is contradictory with other studies, which indicated that diets containing high amounts of MUFA can cause small amounts of weight loss [11]. Nut consumption, in general, has been associated with decreases in BMI [26,27]. In the Adventist Health Study, Fraser et al. reported a statistically significant negative association between consumption of nuts and BMI in a cohort of 31,200 subjects from California. [26,27]. This study showed that those who ate nuts more frequently were leaner than those who infrequently consumed nuts [26,27]. Our study may not have seen these same differences due to the small dosage of pecans used or the fact that pecans were the only source of nuts in our study.

Data gathered from the Vioscreen FFQ suggests that there are significant correlations present between certain micronutrients and FBG levels for individuals with overweight/obesity. Additionally, there are significant correlations between certain micronutrients tested and LDL cholesterol. These results indicate that increasing the amount of dietary magnesium, MUFA, and SFA may be a valid dietary intervention for lowering blood sugar levels. A noteworthy finding of this study was the negative relationship between FBG and MUFA (*r* = −0.61, *p* = 0.01), as well as SFA (*r* = −0.58, *p* = 0.01). This finding supports the theory of a high fat, low carbohydrate diet as an intervention for the maintenance of blood glucose levels in those with insulin resistance. It is particularly interesting that increased proportions of dietary SFA were associated with lower FBG levels, considering current 2026 U.S. Dietary Guidelines suggest limiting saturated fat in the diet [28]. The researchers of this study are unaware of any other literature finding an association between higher saturated fat intake and lower FBG levels. One study did however find that increased saturated fatty acid intake increased gastric inhibitory polypeptide and production of insulin which may be a plausible mechanism for the findings in this investigation [29]. Additional findings from the FFQ results in our study indicate that increasing dietary intake of iron, selenium, magnesium, zinc, MUFA, and PUFA correlated significantly to reduced levels of LDL cholesterol. Increased SFA and dietary cholesterol were also significantly associated with reduced LDL cholesterol; however, this is counterintuitive based on the present understanding of lipoprotein metabolism.

The results of the cytokine levels in the serum suggest that 1.5 oz of pecans added to the regular diet of participants may not lower pro-inflammatory cytokines nor raise anti-inflammatory cytokine levels to be detectable in the serum of the participants. The results also suggest that the methods used were unable to detect changes in the serum cytokines analyzed. One particular challenge is that since all of our participants were within the expected normal ranges for the circulating cytokines we measured (IL-6 ≤ 5.3 pg/mL, IL-10 ≤ 2.9 pg/mL, and TNF-α ≤ 15.4 pg/mL), their levels were overall rather low and/or near the levels of detection (IL-6 = 0.626 pg/mL, IL-10 = 3.9 pg/mL, and TNF-α = 1.7 pg/mL) [23]. This is to be expected among a relatively healthy group of people, however this limits the dynamic range by which to distinguish any differences and any shifts would be expected to be rather small. Although cytokines play roles systemically in the body, the shift in expression of them may also be a larger factor of the local tissue as opposed to systemic circulation. The immune system itself is heavily regulated to control the level of inflammation, so a dietary intervention of this size may not have been large enough to detect statistically significant changes in our population. The number of participants is also a challenge as the total number of participants for serum measurements was 19 and not all participants finished the study.

It is noteworthy that there were some limitations to the current preliminary study that may have affected results. First, the study had a relatively small sample size with 22 participants. We also did not use an isocaloric control, which may increase bias in participants. Another potential limitation to this study is the use of the FFQ to collect dietary data from participants. Shim JS, Oh K, and Kim HC note that limitations to this tool include the use of closed-ended questions and the need for participants to accurately interpret what is being asked by each question [30]. Furthermore, it is important to note the Food Frequency Questionnaire was a self-completed survey and holds the potential for recall inaccuracies and answer bias [30]. Lastly, with the inclusion of females up to age 65 in the current study, there may be some variation in results between pre- and post-menopausal women. In future studies, it will be important to assess hormonal status along with cardiometabolic factors.

The results from the present study suggest a possible relationship between certain consumed micronutrients and various metabolic parameters. Specifically, there was a strong positive correlation between vitamin D intake and BMI measurements. This suggests that increased intake of foods high in vitamin D, such as dairy products, may result in higher BMIs. These findings should be further investigated in future studies. Another limitation to this study could have been that participants were entirely from the Midwest region, which may have influenced the type of food that participants consumed, such as a potential increase in dairy products. Due to several limitations, observations made in this study may not be applicable to other populations. Through these findings it can be concluded that an intervention of consuming 1.5 oz of pecans for 8 weeks did not significantly change measured metabolic parameters nor serum cytokine levels. Although these results were not what was hypothesized, they do lead to a need for further studies. Future work should include a longer intervention period, a larger cohort study, and potentially a larger dosage of pecans to increase the amount of MUFA. Furthermore, additional cytokines could be investigated and more sensitive techniques for measurement to distinguish potentially small changes. Adipose tissue samples could also serve as a mechanism to measure local inflammatory status. Such modifications may allow future studies to build on this initial study and truly determine if a pecan intervention providing MUFA can influence cardiometabolic parameters and/or serum cytokine levels in humans.

## Figures and Tables

**Table 1:** Participant Demographics with normal values delineated. Mean baseline values ± SEM.

Participant Demographics (*n* = 22)	
Variable	Average ± SEM
BMI 18.5–24.9 (kg/m^2^)	32.2 ± 1.2
FBG 60–99 (mg/dL)	95.1 ± 2.8
Total Calories 1200–2000 (kcal)	1536 ± 229
Total Cholesterol < 200 (mg/dL)	207.9 ± 7.0
HDL (mg/dL)	58 ± 4.6
LDL (mg/dL)	127.1 ± 6.1
Triglycerides(mg/dL)	133 ± 10.1
Iron (mg)	14.2 ± 1.3
Selenium (mcg)	115 ± 12.1
Magnesium (mg)	330.5 ± 23.5
Vitamin E (IU)	17.4 ± 2.5
Zinc (mg)	12.5 ± 1.2
MUFA (g)	28.8 ± 2.9
SFA (g)	27.1 ± 2.7
PUFA (g)	15.9 ± 1.5

**Table 2:** Mean baseline values between the control group and pecan intervention group. There was no significant differences between these groups.

Baseline Averages ± SEM	Control (*n* = 11)	Experiment (*n* = 11)	*p*-Value
BMI	33.1 ± 1.7	32.0 ± 1.2	0.45
Waist:Hip Ratio	0.91 ± 0.02	0.89 ± 0.02	0.53
Body fat%	42.48% ± 0.03	43.28% ± 0.03	0.50
Visceral Fat	208.15 ± 26.2	221.77 ± 29.1	0.34

**Table 3:** Mean post-intervention values between the control group and pecan intervention group. There was no significant differences between these groups.

Body Composition Post-Intervention Values			
	Control (*n* = 11)	Experimental (*n* = 11)	*p*-Value
BMI	32.7 ± 9.1	31.5 ± 8.8	0.55
Waist:Hip Ratio	0.89 ± 0.25	0.86 ± 0.24	0.33
Body fat%	40.88 ± 0.11	39.99 ± 0.11	0.82
Visceral Fat	241.2 ± 66.91	225.6 ± 62.57	0.69

**Table 4:** Baseline (B) and Post-Intervention (PI) values. The *p*-value is shown at baseline and endpoint between the two groups.

Lipid Panel and Fasting Blood Glucose Baseline and Post-Intervention Values			
	Control (*n* = 11)	Experimental (*n* = 11)	*p*-Value
Total Cholesterol (mg/dL)	B: 202.76 ± 31.48	B: 215.25 ± 30.18	B: 0.32
	PI: 215.92 ± 34.83	PI:233.69 ± 40.13	PI: 0.23
HDL (mg/dL)	B: 57.17 ± 21.65	B: 56.67 ± 21	B: 0.95
	PI: 60.54 ± 19.93	PI: 60.08 ± 19.19	PI: 0.95
LDL (mg/dL)	B: 122.36 ± 31.78	B: 133.4 ± 24.66	B: 0.35
	PI:132.58 ± 31.59	PI:142.69 ± 37.96	PI: 0.48
VLDL (mg/dL)	B: 33.9 ± 33.11	B:30.2 ± 10.42	B: 0.72
	PI: 32.98 ± 30.12	PI: 30.9 ± 17.12	PI: 0.84
Triglycerides (mg/dL)	B:126.64 ± 42.62	B:140.91 ± 59.87	B: 0.53
	PI: 123.83 ± 34.20	PI: 153.46 ± 69.19	PI: 0.19
Fasting Blood Glucose (mg/dL)	B:97.08 ± 16.61	B:100.08 ± 18.72	B: 0.67
	PI: 93.30 ± 8.14	PI: 103.23 ± 20.47	PI: 0.12

**Table 5:** R value comparing fasting blood glucose to FFQ-determined nutrients.

Correlation of Fasting Blood Glucose to the Corresponding Micronutrient		
Micronutrient	r	*p*-Value
Dietary Cholesterol	0.12	0.57
Iron	−0.23	0.75
Selenium	−0.09	0.51
Magnesium	−0.2	0.01
Vitamin E	−0.07	0.89
Zinc	−0.12	0.6
MUFA	−0.16	0.05
SFA	−0.2	0.03
PUFA	−0.03	0.89

**Table 6:** R value comparing LDL cholesterol to FFQ-determined nutrients.

Correlation of LDL Cholesterol to Corresponding Micronutrient		
Micronutrient	r	*p*-Value
Dietary Cholesterol	−0.34	0.02
Iron	−0.13	0.01
Selenium	−0.25	0.004
Magnesium	0.04	0.01
Vitamin E	0.07	0.77
Zinc	−0.21	0.004
MUFA	−0.18	0.007
SFA	−0.06	0.03
PUFA	−0.34	0.0001

**Table 7:** Shows the correlations and levels of significance between certain nutrient intakes and cardio- metabolic measures.

Correlational Relationships Between Dietary Intake and Cardio-Metabolic Parameters						
	BMI	Total Cholesterol	HDL	LDL	VLDL	Blood Glucose
Correlation of Vitamin A and	0.19	−0.21	0.08	−0.10	−0.45	−0.08
*p*-value	0.47	0.32	0.91	0.69	0.05	0.64
Correlation of Vitamin C and	−0.04	−0.03	0.17	0.04	−0.44	−0.21
*p*-value	0.88	0.92	0.4	0.89	0.04	0.37
Correlation of Vitamin D and	0.71	−0.35	−0.48	−0.13	0.19	0.37
*p*-value	0.0004	0.09	0.007	0.6	0.3	0.11
Correlation of Sodium and	0.21	−0.36	−0.12	−0.16	−0.35	−0.24
*p*-value	0.42	0.09	0.45	0.49	0.0003	0.24
Correlation of Dietary Fiber and	0.19	−0.11	0.13	−0.05	−0.38	−0.27
*p*-value	0.74	0.64	0.6	0.84	0.09	0.23

## Data Availability

The data presented in this study are available on request from the corresponding author.
